# Task and Non-task Brain Activation Differences for Assessment of Depression and Anxiety by fNIRS

**DOI:** 10.3389/fpsyt.2021.758092

**Published:** 2021-11-05

**Authors:** Dan Wen, Xuenan Lang, Hang Zhang, Qiqi Li, Qin Yin, Yulu Chen, Yong Xu

**Affiliations:** ^1^First Hospital of Shanxi Medical University, Taiyuan, China; ^2^Department of Psychiatry, First Hospital/First Clinical Medical College of Shanxi Medical University, Taiyuan, China; ^3^Department of Mental Health, Shanxi Medical University, Taiyuan, China

**Keywords:** functional near-infrared spectroscopy, non-task, verbal fluency task, anxiety, depression

## Abstract

Diagnosis and treatment of the patients with major depression (MD) or the combined anxiety and depression (A&D) depend on the questionnaire, sometimes accompanied by tasks such as verbal fluency task (VFT). Functional near infrared spectroscopy (fNIRS) is emerging as an auxiliary diagnostic tool to evaluate brain function, providing an objective criterion to judge psychoses. At present, the conclusions derived from VFT or rest (non-task) studies are controversial. The purpose of this study is to evaluate if task performs better than non-task in separating healthy people from psychiatric patients. In this study, healthy controls (HCs) as well as the patients with MD or A&D were recruited (*n* = 10 for each group) to participate in the non-task and VFT tasks, respectively, and the brain oxygenation was longitudinally evaluated by using fNIRS. An approach of spectral analysis is used to analyze cerebral hemoglobin parameters (i.e., Oxy and Deoxy), characterizing the physiological fluctuations in the non-task and task states with magnitude spectrum and average power. Moreover, the standard deviation of oxygenation responses during the non-task was compared with the peak amplitude during the task, with the aim to explore the sensitivity of the VFT task to brain activation. The results show that there is no significant difference (p > 0.05) among the three groups in average power during non-task. The VFT task greatly enhanced the magnitude spectrum, leading to significant difference (p < 0.05) in average power between any of two groups (HC, MD, and A&D). Moreover, 40% patients with A&D have an intermediate peak (around 0.05 Hz) in the magnitude spectrum when performing the VFT task, indicating its advantage in characterizing A&D. We defined a rate of the non-task standard variation to the task peak amplitude (namely, SD-to-peak rate) and found that this rate is larger than 20% in 90% of the MD subjects. By contrast, only 40% HC subjects have an SD-to-peak rate larger than 20%. These results indicate that the non-task may not be sufficient to separate MD or A&D from HC. The VFT task could enhance the characteristics of the magnitude spectrum, but its intensity needs to be elevated so as to properly explore brain functions related to psychoses.

## Background

Depression and anxiety are two psychoses that affect millions of people in the world, and their comorbidity rate is high (1). Clinical symptoms and functional impairment are more severe in patients with the combined anxiety and depression (A&D) (2), and the major consequences include emotional, cognitive, and somatic symptoms. It has been found that the brain functions of A&D are different from either depression or anxiety alone. Patients with depression have the suppressed functions of brain regions that regulate emotion and cognition, thus exhibiting the relevant clinical symptoms (3, 4). By contrast, the alterations in cerebral cortex function occur in specific regions for A&D patients, particularly in the prefrontal lobe and temporal lobe, affecting execution, language, memory, and attention, as evidenced by the declined cognitive control ability (5).

Rapid advances in neurological imaging technologies, such as functional magnetic resonance imaging (fMRI) and electroencephalography (EEG), permit observation of the changes in both brain structure and function. Although fMRI could obtain functional parameters such as oxy-hemoglobin index, the high cost precludes its routine use in psychiatric studies. In addition, fMRI measurement is strictly limited by body posture, making it difficult to perform verbal or cognitive tasks simultaneously. EEG provides rapid assessment of neural activity in the cerebral cortex and has been widely utilized in cognition and autonomic control. However, EEG does not directly reflect brain metabolism.

Functional near-infrared spectroscopy (fNIRS) is a non-invasive, functional neuroimaging technique that could assess brain function by quantifying cerebral oxygenation at the microvasculature level (6–8). After more than 40 years of development, this technology has been used for evaluation of cerebral function (9–11) and extended to the clinical field of psychiatry, as one of the objective modalities for probing psychoses (12). fNIRS is sensitive to external stimulation or events; it allows for longitudinal monitoring of the changes in brain metabolism. Hence, fNIRS is frequently adopted for brain functional assessment when various tasks are performed (13).

The non-task (i.e., doing nothing) is a resting state that is easy to implement. The spontaneous brain activity during non-task period can be used as a baseline reference for brain activation (14). In EEG studies, the features in the resting state were found to be associated with a variety of neuropsychiatric disorders (15). fMRI can explore brain function by measuring the temporal variations of blood oxygenation level dependence (BOLD) as well as the fluctuations in resting spontaneous neural activity (16–18). Besides these, the spectral analysis of fNIRS permits the assessment of the autoregulation of brain function. The non-task does not generate motion artifacts and the obtained fNIRS signal is more reliable, with minimal signal noise. Recently, minor brain damage in the early stage of Alzheimer's was detected from the non-task EEG-fNIRS data (19). On the other hand, however, non-task does not challenge the brain function, which can only be realized by task paradigm (20).

The verbal fluency task (VFT) is a simple task that is widely used to assess language extraction and processing abilities (21, 22). fNIRS was also applied on the VFT task to distinguish different types of psychoses (23). In some studies, major depression (MD) patients were found to have the reduced responses in the orbitofrontal cortex (OFC) and frontal cortex (24), and the activation level is associated with the severity of MD symptoms (25). The schizophrenia patients were also found to have the reduced responses in the dorsolateral prefrontal cortex (DLPFC) (26), and bipolar disorder patients have the same performance in the left inferior frontal gyrus (IFG) (27). Under the VFT task, the prefrontal lobe is well activated (28), when compared with the non-task. In some fNIRS studies, it was found that the damaged cerebral cortex can be detected from both the non-task and the task. Hence, fNIRS technology is sensitive to both cognitive tasks and static state. However, task and non-task have not been extensively compared thus far.

Therefore, the comparison of cortex oxygen responses between VFT task and non-task will contribute to the detection of psychoses such as MD and A&D. In addition, an in-depth analysis of the response pattern of brain oxygenation variables in healthy and psychiatric populations would effectively evaluate and further optimize fNIRS for the detection of psychoses (29).

## Materials and Methods

### Participants

In this study, 10 patients with MD and 10 patients with A&D diagnosed according to the Diagnostic and Statistical Manual of Mental Disorders-V (DSM-V) (30) criterion were recruited at the outpatient clinic of the Department of Mental Health, First Hospital of Shanxi Medical University. The diagnosis was conducted by three experienced physicians according to the consistent criteria. Ten healthy controls (HCs) were recruited from the local community. The subjects were in the age range between 15 and 55 years. The average age of the subjects for MD, A&D, and HC, represented by mean ± standard derivation, are 29.8 ± 11.8, 30.0 ± 11.4, and 31.8 ± 11.1, respectively. Every two of the three groups are aged-matched (*p* > 0.05). The time-course changes in cerebral cortex oxygenation during the VFT task and non-task were measured and analyzed among the three groups of subjects. All subjects have education level of high school level or above, and those who have neuropathy, severe physical diseases, substance abuse, or high suicide risk were excluded. The study was approved by the Ethics Committee of the First Hospital of Shanxi Medical University. After the study design was explained, all of the participants signed the consent form.

### Methods

#### fNIRS Data Acquisition

The oxygenation data were collected by the Hitachi ETG-4100 fNIRS instrument (52CH) at the First Hospital of Shanxi Medical University. The subjects completed the non-task and VFT protocol individually. Prior to experiments, the subject wore a helmet embedded with numerous near-infrared light sensors, covering the prefrontal region and both temporal lobes. The near-infrared light sensors were placed over the scalp according to the 10–20 international system. Each channel of the near-infrared signals is collected from a source–detector (S–D) pair, at the separation of 3.0 cm. A total of 11 S–D channels are located on the prefrontal lobe, and 20 S–D channels are located on the right and left temporal lobes. For each S–D channel, the near-infrared light is injected from the two laser sources (695 and 830 nm) alternately into the human scalp. The reflected signals from the brain cortex are collected by the optical detector and used to calculate the oxygenation responses. The laser power (8 mW) is sufficiently low so as to be approved for clinical usage. Both prefrontal lobe and temporal lobes are reported to be activated to the VFT task. For all the subjects, the oxygenation data obtained from each channel were averaged over the prefrontal lobe and temporal lobes, yielding the average responses to the VFT task and non-task respectively.

Firstly, the 160-s non-task data were collected from all subjects. The non-task requires the participant to sit in a chair, open their eyes and look forward, and repeatedly count 1 through 10, with the thumbs and index fingers of both hands performing counter-finger movements in rhythm until the end of the task (31). The subject was then asked to rest for 5 min. Subsequently, 160-s VFT task data were collected from all subjects. The fNIRS signals were collected for 30 s as the baseline prior to the task. During the VFT task, the subject was asked to construct the phrase with the simple words such as sky, earth, and human, lasting for 60 s (32, 33). The post-task fNIRS signals were collected for 70 s, repeatedly counting from 1 through 10. The cerebral oxygenation variables at each channel, including concentration of oxygenated hemoglobin (Oxy) and deoxygenated hemoglobin (Deoxy), were calculated from fNIRS signals according to the Beer–Lambert law. These variables at different channels were then averaged over the prefrontal region and both temporal lobes, yielding the cortex responses to the task or non-task.

### Data Analysis

The spectral analysis was used to investigate the time-course oxygenation variables (Oxy and Deoxy) curves, yielding the relationship between frequency and the magnitude of spectrum (34–36). This spectrum relationship is to characterize the oxygenation oscillations of the three populations (MD, A&D, and HC). Specifically, the Oxy/Deoxy data were collected using the fNIRS instrument at the sample time of 0.1 s, leading to a total of 1,600 data points over the 160-s measurement period. These time-course curves were preprocessed by a Hanning window to reduce the effect of spectral leakage. Then, fast Fourier transform (FFT) was applied to transform the time-course curve into the frequency domain, from which the magnitude spectrum of the same length was obtained over the 0.01–16.0-Hz range, at the sample rate of 0.01 Hz. The average power, defined as the average of magnitude square over the whole spectrum (0.01–16.0 Hz), was calculated for each individual. In addition, the rate between the standard deviation of the Oxy/Deoxy curve during non-task and the peak amplitude during VFT task was calculated, in order to explore the sensitivity of the VFT task to brain activation. Furthermore, the Bland–Altman analysis is adopted to assess the dispersion of the intra-group data, and the threshold is set as 1.96 times of standard deviation (SD).

## Results

### Comparison of the Rate of Brain Activation Between Non-task and VFT Task

**Figure 1:**
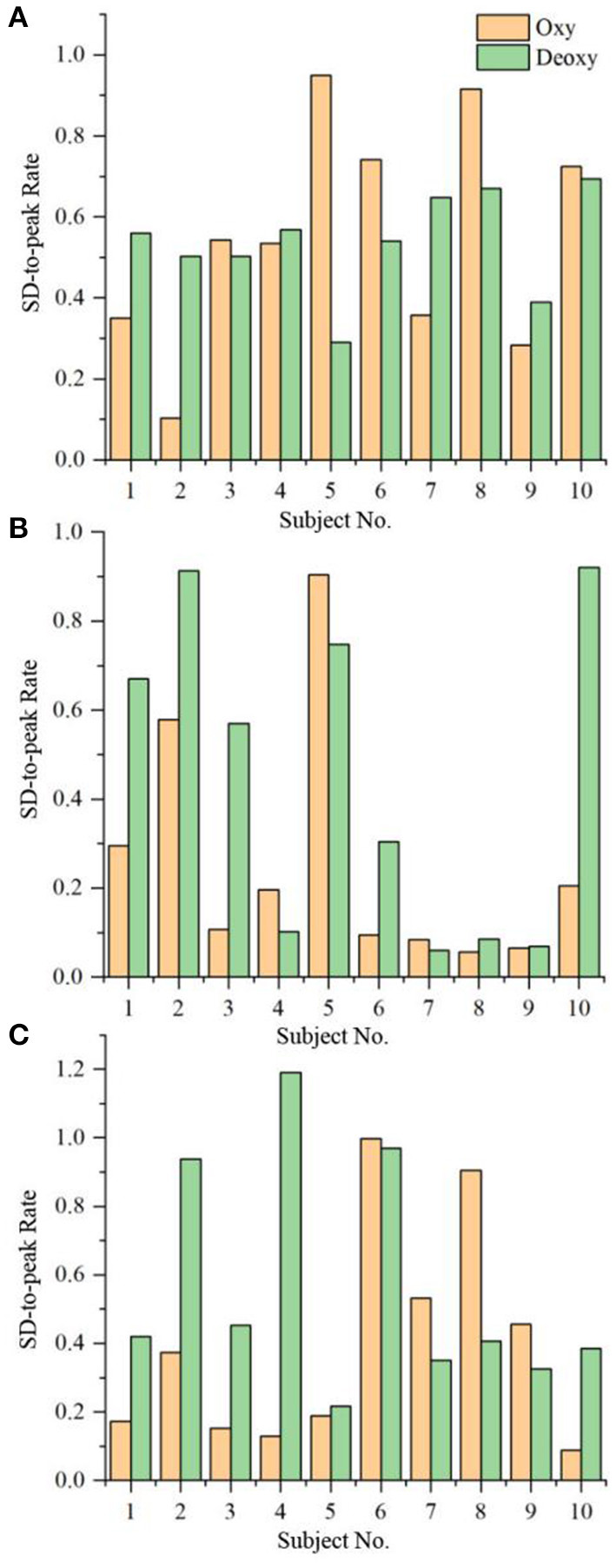
The rate of the Oxy/Deoxy standard deviation during non-task to the maximal change (i.e., peak amplitude) during VFT task for the three groups, i.e. **(A)** MD, **(B)**, HC and **(C)** A&D.

Figure 1 shows the rate of the Oxy/Deoxy standard deviation during the non-task to the maximal change (i.e., peak amplitude) during the VFT task, namely, SD-to-peak rate, for the three groups. It can be seen that 90% of the MD patients had an SD-to-peak rate larger than 20% (Figure 1A), while 40% of the HCs have a rate larger than 20% (Figure 1B). As for the A&D patients, 60% of the group have a rate larger than 20% (Figure 1C). Additionally, it is also found that the intra-subject variability of this rate was large in each group of subjects, and no significant differences were found between groups. Regression analyses indicate that the age is not relevant (*p* > 0.05) to the NIRS outcomes (i.e., Oxy and Deoxy).

### Distribution in the Degree of Brain Activation in the Non-task State

**Figure 2:**
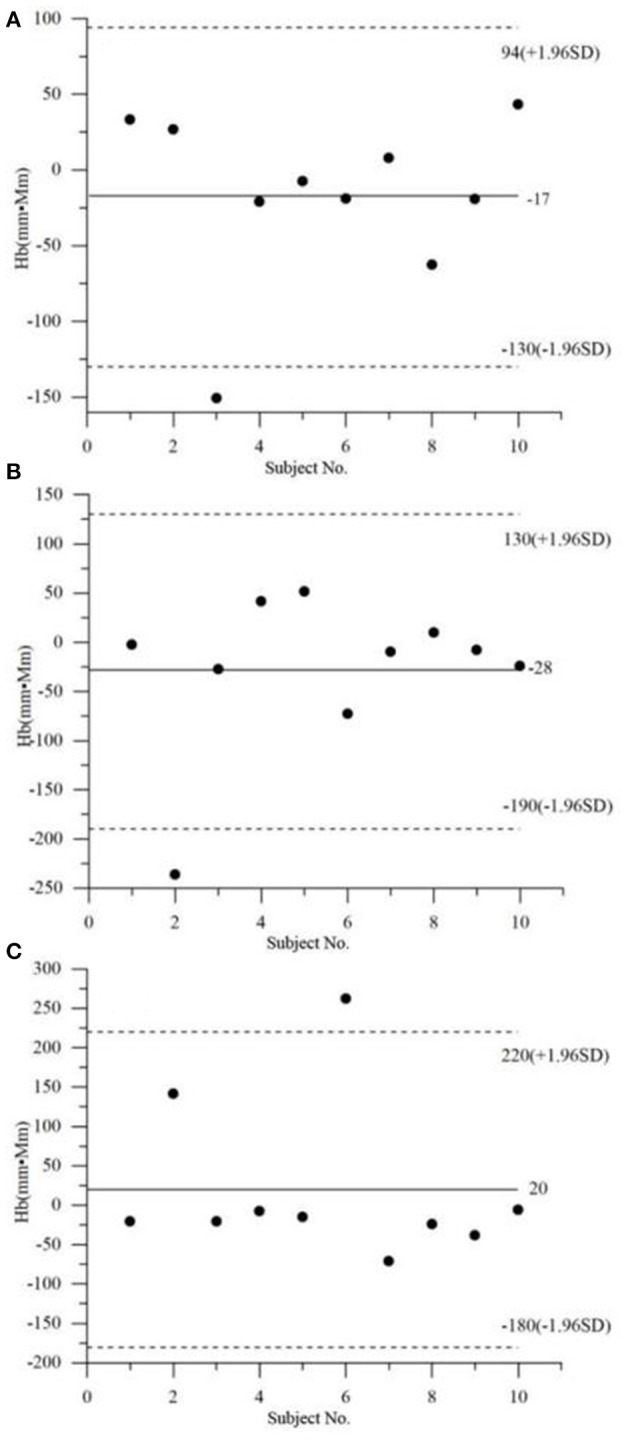
Bland–Altman analysis of the Oxy integral values (i.e., area of the Oxy curve) by the non-task, in the three groups, i.e., **(A)** MD, **(B)** HC, and **(C)** A&D.

Figure 2 exhibits the Bland–Altman analysis of the Oxy integral values by the non-task. As seen clearly, 10% (1/10) of the data points are outside the limit of 1.96 times the standard deviation, regardless of the group (i.e., MD, HC, or A&D). The Bland–Altman analysis of the Deoxy exhibits a similar conclusion. These results indicate that the healthy and mental-disordered populations have similar intra-subject variability.

### The Difference Between Non-task and VFT Task in the Oxy Magnitude Spectrum

**Figure 3:**
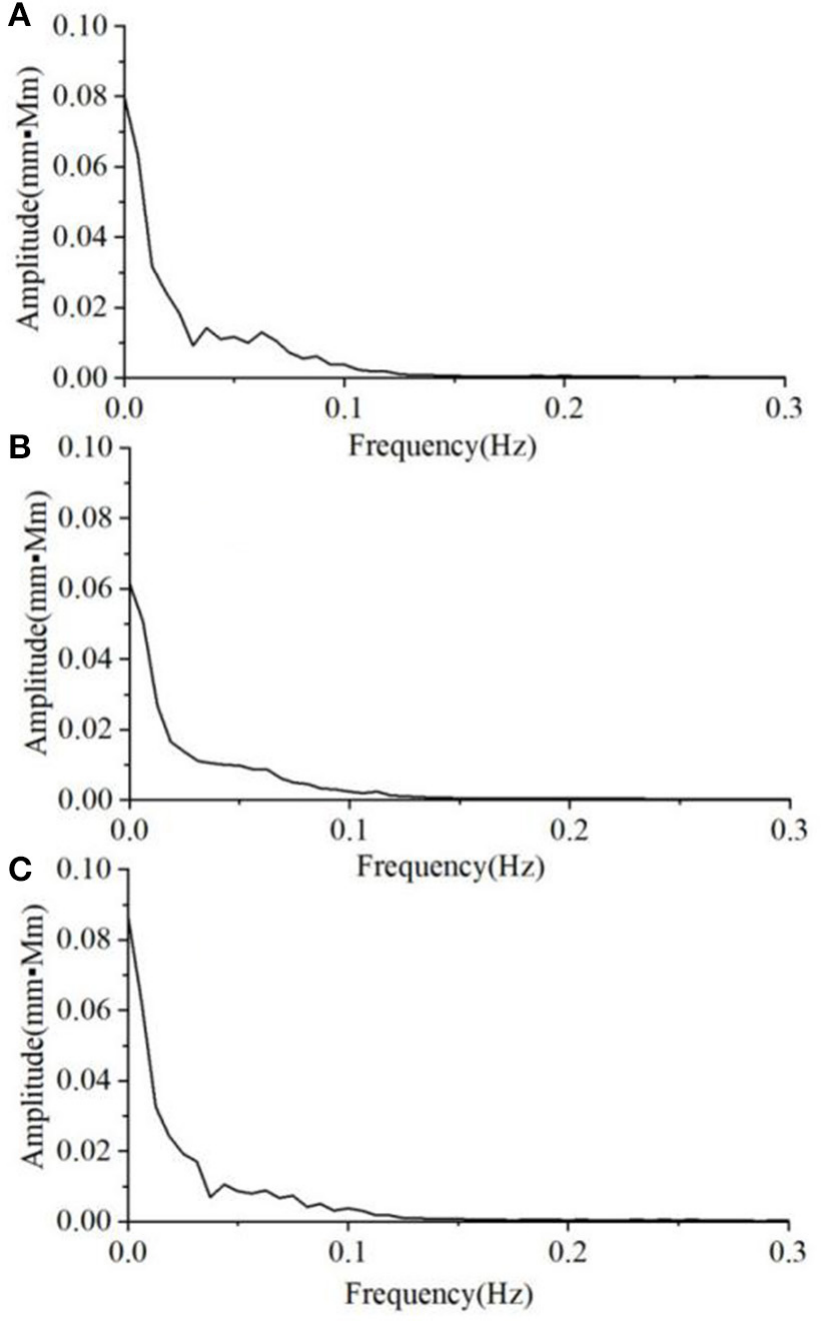
The average magnitude spectrum of the Oxy time-course curves during non-task, in the three groups, i.e. **(A)** HC, **(B)** MD, and **(C)** A&D. Note that the magnitude is almost zero at the range of 0.3–16.0 Hz and not shown in the figure.

**Figure 4:**
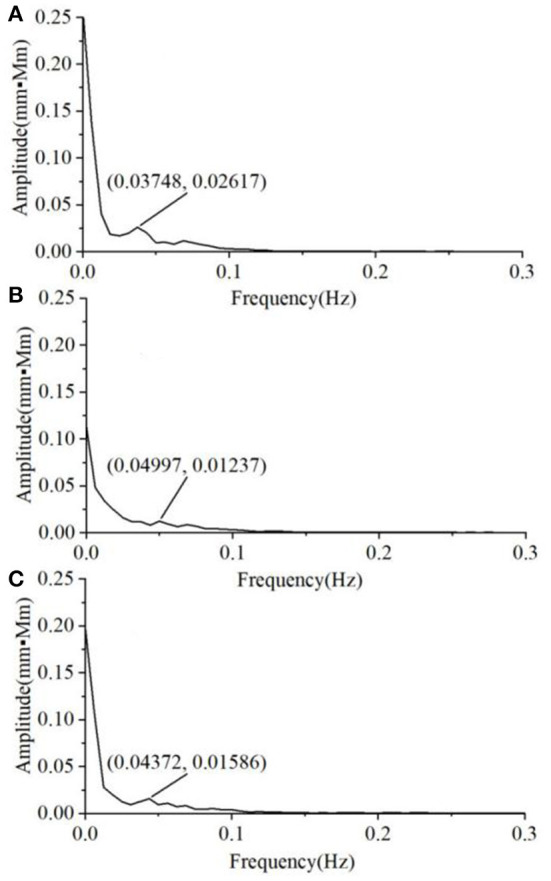
The average magnitude spectrum of the Oxy time-course curves during the VFT task in the three groups, i.e. **(A)** HC, **(B)** MD, and **(C)** A&D. Note that the magnitude is almost zero at the range of 0.3–16.0 Hz and not shown in the figure.

The average magnitude spectra of Oxy time-course curves during non-task and VFT task in the three groups are shown in Figures 3, 4, respectively. Under non-task, it can be seen that the magnitude decreased with the increase in frequency (Figure 3). The groups in magnitude order from largest to the smallest were HC, A&D, and MD. The average magnitude during the VFT task were substantially higher than that during the non-task (around twice), while the order remained the same (i.e., HC, A&D, and MD).

The Oxy average power (represented by mean ± standard derivation) during non-task is 4.8± 9.3, 1.7±1.3, and 3.2±5.1 (10^−2^·mm·Mm)^−2^ for HC, MD, and A&D, respectively, and no significant difference (*p* > 0.05) was found among the groups. This average power was greatly enhanced during VTF task, reaching 14.0 ± 9.8, 2.9 ± 3.4, and 6.7 ± 4.5, respectively. Furthermore, a significant difference (p < 0.05) was found between any of the two groups (i.e., HC, MD, and A&D).

### Characteristics of the Typical Magnitude Spectrum in the Task State

**Figure 5:**
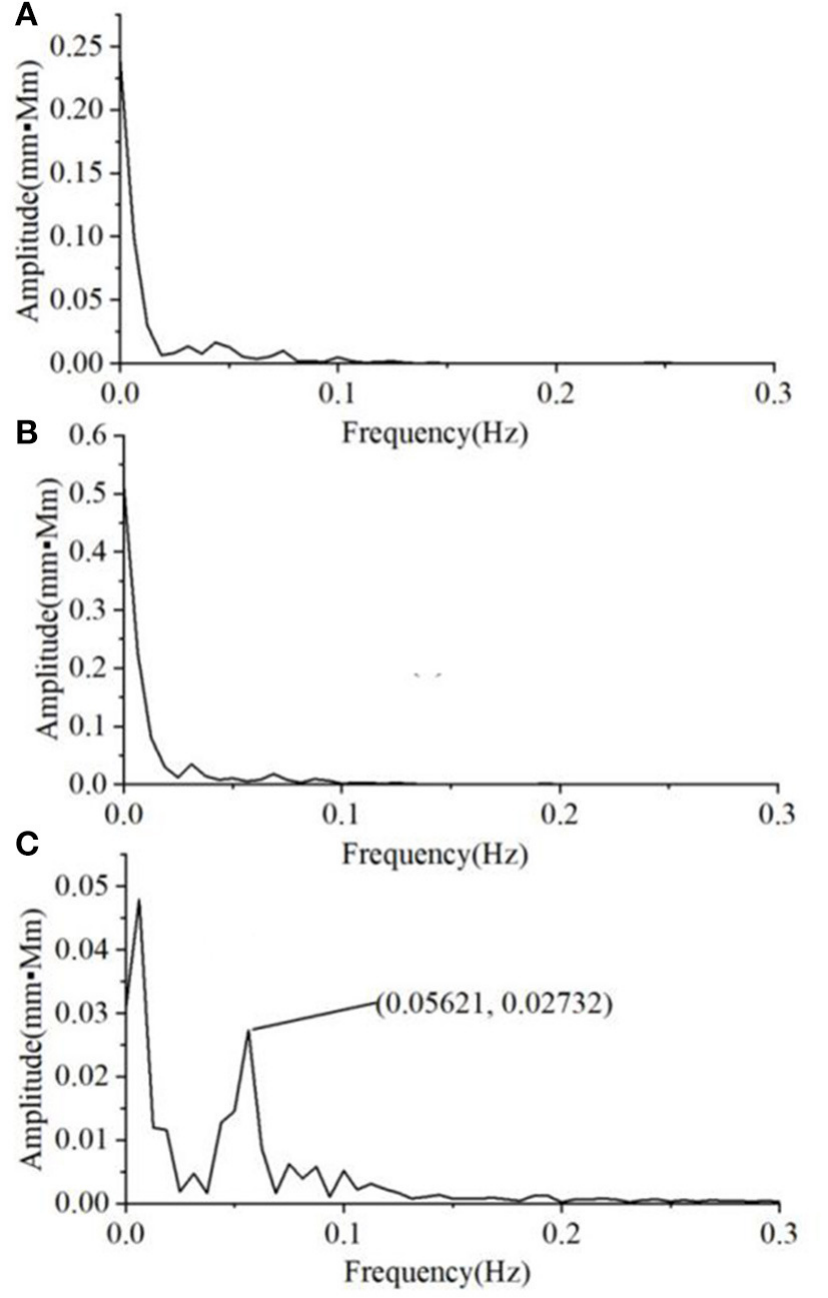
The typical Oxy magnitude spectrum curve from a representative individual in each of three groups, i.e. **(A)** HCS, **(B)** MD, and **(C)** A&D. Note that the magnitude is almost zero at the range of 0.3–16.0 Hz and not shown in the figure.

We also found that the variability of the Oxy magnitude spectrum curve at the non-task state is small, regardless of the group. Hence, the average magnitude spectrum curve would well-represent the individual responses. By contrast, there is much larger inter-subject variability in the Oxy magnitude spectrum when the VFT task was performed. Figure 5 shows the typical Oxy magnitude spectrum curve from a representative individual in each group. The MD exhibits the magnitude spectrum curve similar to that of HC, with a small peak value around 0.05 Hz (Figures 5A,B). By contrast, four of the 10 A&D showed a strong intermediate peak around 0.05 Hz (Figure 5C). This is because not all of the A&D patients had such strong intermediate peak, whose frequency also varied in a range (i.e., not the same frequency). Therefore, the average spectrum curve of the A&D population did not clearly exhibit the strong intermediate peak. Nevertheless, none of the HC and MD groups were found to have such a special feature.

## Discussion

fNIRS is an easy-to-use and low-cost technology for assessing brain function that is associated with cerebral metabolism. This technology has also been used as the auxiliary tool for diagnosis of psychoses (37). For example, the fNIRS was used to assess the autoregulation capacity of the brain cortex, which may be affected by psychoses. The protocols of non-task or task were adopted for this assessment (38, 39). The specificity and sensitivity of psychiatric diagnosis relies on several factors including the detection techniques, experiment protocols, and stimulus intensity. At present, the conclusions derived from VFT or rest (non-task) studies are controversial, and no oxygenation parameters were claimed to diagnose the psychiatric diseases. Several factors might account for this reason, such as the task intensity and duration for evoking the brain cortex, the intra-subject variability, and the oscillation characteristics related to the mental disorders. This study is designed to explore if the VFT task is strong enough to challenge the brain cortex, when compared with the rest state. We also investigated if the oscillation characteristics, which are generally evaluated by the rest state, could be affected by the VFT task.

A total of 30 subjects were recruited to participate in this study, with 10 for each group (HC, MD, and A&D). The sample size (*n* = 10) might not be large enough to observe the statistical difference. Nevertheless, the similar sample size (*n* = 6–12) is often reported in the NIRS studies exploring the brain cortex functions, along with statistical analysis results (40–44). The sample size is limited to the subject's availability and willingness to participate in the study. More subjects will be recruited and the advanced analysis algorithms will be explored, which is one objective of our future works.

A comparative study on brain oxygenation response by task and non-task was conducted on the 30 subjects. VFT was chosen as the task model because it is most widely used in psychiatric evaluation, and the changes in the brain oxygenation were found during the task period. Moreover, we assessed the task and non-task states in both amplitude change and spectrum. Previous studies have shown that MD patients have the reduced brain metabolism and task responses, indicating a cognitive impairment (45). Furthermore, the insufficient performance of MD patients was also observed when conducting the VFT task (46).

In order to evaluate the brain oxygenation activation by the VFT task, an index of SD-to-peak was calculated for each subject. We found that 90% of MD patients have a rate larger than 20%, indicating that MD is associated with the deficiency in brain oxygen activation by the VFT task, which would be a marker of depressive state. By contrast, this rate is larger than 20% in 40% HC and 60% A&D populations, respectively (Figure 1), indicating that majority of A&D responded positively to the VFT task. These observations exhibit that fNIRS has the potential to separate the depression through the brain oxygenation activation, if proper task is implemented.

With the approach of Bland–Altman to analyze the distribution of the Oxy integral values in MD, HC, and A&D, we found that 10% (1/10) of the data points are outside of the 1.96 times the standard deviation (Figure 2), regardless of the group. This observation demonstrates that MD, HC, and A&D are similar in terms of individual data distribution.

As for the spectral analysis, we found that the magnitude is decreased with the increase in frequency. For each individual, the magnitude spectrum was mainly focused at 0.05–0.1 Hz, indicating low-frequency oscillations in the spontaneous state. Under the non-task protocol, no significant difference was found among the three groups (Figure 3), indicating that static status would not alter the characteristic of low-frequency oscillation.

When performing the VFT task, we found that the magnitude of spectrum was substantially enhanced for all the individuals, with HC being the highest, A&D the second, and MD the lowest. In the past, it is difficult to separate MD and A&D by using fNIRS or other neuroimaging modalities. In this study, 40% A&D patients were found to show a strong intermediate peak (around 0.05 Hz) in the magnitude spectrum when performing the VFT task, while this strong intermediate peak was not found in HC and MD populations. Although the underlying mechanism for this difference is unclear, this observation is beneficial for future diagnosis of the A&D disease.

Our spectral analyses of oxygenation data verified this hypothesis, i.e., the magnitude during the VFT task is much higher than that during the non-task status. We also observed that the Oxy average power has no significant difference among the three groups during non-task. By contrast, a significant difference was found between any of the two groups. These outcomes indicate that the parameter of average power, when enhanced by the VFT task, also characterizes well the diseased populations.

The aim of the repeated number counting and rhythm finger movement is to enforce the subjects to concentrate on this tedious task and minimize the intra-subject variability resulting from random thinking. According to the literature (47), the finger movement would primarily evoke the activities in the parietal lobe, rather than the prefrontal lobe and temporal lobes that were measured with fNIRS in this study. Hence, the fNIRS signals originating from finger movement can be considered as a small portion of the baseline data.

Nevertheless, the baseline data (including those from number counting and finger movements) may also affect the data analyses. The potential solution for minimizing the baseline effect would be increasing the task intensity (e.g., VFT duration) as well as utilizing the advanced algorithms (e.g., independent component analysis—ICA) to separate the baseline signals, which will be one objective of our future works.

As the summary, the outcomes derived from this study demonstrate that the standard VFT (60 s) might not be able to evoke sufficient oxygenation changes in the brain cortex, as evidenced by the >20% SD-to-peak rate in the majority of subjects, especially in MD patients. We also found that the VFT might enhance the oscillation characteristics of Oxy curves. The intra-subject variability is similar among the three groups. All of these discoveries have not been reported in the previous studies. We believe that the fusion of multiple parameters, rather than the single parameter, would better localize the target diseases (MD or A&D), along with the advanced algorithm (e.g., artificial intelligent algorithm). The conclusion derived from this study might be helpful in future exploring the advanced diagnostic approaches.

## Conclusions

To conclude, fNIRS is an easy-to-use technology for longitudinally monitoring brain function (48), and its efficiency for psychosis diagnosis relies on the physiological protocol. In this study, we used fNIRS to compare between task and non-task states in brain oxygenation activation and to explore the characteristic markers of MD and A&D. We found that the MD is associated with the deficient activation in brain oxygenation, evidenced by the relatively large SD-to-peak rate. In addition, A&D is characterized by the intermediate peak in spontaneous low-frequency oscillations. Nevertheless, the precise diagnosis of MD and A&D is negatively affected by the intra-subject variability. The optimal tasks, with small intra-subject variability and the enhanced oxygenation activation, need to be developed to further explore the brain functions associated with psychoses (13, 49).

## Data Availability Statement

The original contributions presented in the study are included in the article/supplementary material, further inquiries can be directed to the corresponding authors.

## Ethics Statement

The studies involving human participants were reviewed and approved by the Ethics Committee of the First Hospital of Shanxi Medical University. Written informed consent to participate in this study was provided by the participants' legal guardian/next of kin.

## Author Contributions

DW and XL conceived of and led on the study design, managed the literature searches, and undertook the statistical analysis, under the supervision of YX, and wrote the first draft. HZ and YC made the subsequent revisions of the manuscript. QL and QY contributed to collecting data. All authors contributed to and have approved the final manuscript.

## Funding

This study was financially supported by the National Natural Science Foundation of China (81971601 and 81701326), the National Key Research and Development Program of China (2016YFC1307004), and the Multidisciplinary Team for Cognitive Impairment of the Shanxi Science and Technology Innovation Training Team (201705D131027).

## Conflict of Interest

The authors declare that the research was conducted in the absence of any commercial or financial relationships that could be construed as a potential conflict of interest.

## Publisher's Note

All claims expressed in this article are solely those of the authors and do not necessarily represent those of their affiliated organizations, or those of the publisher, the editors and the reviewers. Any product that may be evaluated in this article, or claim that may be made by its manufacturer, is not guaranteed or endorsed by the publisher.
